# CrossFuNet: RGB and Depth Cross-Fusion Network for Hand Pose Estimation

**DOI:** 10.3390/s21186095

**Published:** 2021-09-11

**Authors:** Xiaojing Sun, Bin Wang, Longxiang Huang, Qian Zhang, Sulei Zhu, Yan Ma

**Affiliations:** 1 College of Information, Mechanical and Electrical Engineering, Shanghai Normal University, Shanghai 200234, China; 1000479063@smail.shnu.edu.cn (X.S.); qianzhang@shnu.edu.cn (Q.Z.); suleizhu@shnu.edu.cn (S.Z.); ma-yan@shnu.edu.cn (Y.M.); 2 Shenzhen Guangjian Technology Company Ltd., Shanghai 200135, China; longxiang.huang@deptrum.com

**Keywords:** hand pose estimation, convolutional neural network, RGBD fusion

## Abstract

Despite recent successes in hand pose estimation from RGB images or depth maps, inherent challenges remain. RGB-based methods suffer from heavy self-occlusions and depth ambiguity. Depth sensors rely heavily on distance and can only be used indoors, thus there are many limitations to the practical application of depth-based methods. The aforementioned challenges have inspired us to combine the two modalities to offset the shortcomings of the other. In this paper, we propose a novel RGB and depth information fusion network to improve the accuracy of 3D hand pose estimation, which is called CrossFuNet. Specifically, the RGB image and the paired depth map are input into two different subnetworks, respectively. The feature maps are fused in the fusion module in which we propose a completely new approach to combine the information from the two modalities. Then, the common method is used to regress the 3D key-points by heatmaps. We validate our model on two public datasets and the results reveal that our model outperforms the state-of-the-art methods.

## 1. Introduction

Hands plays an important role in the real world as the most direct tools for human comprehension and interaction with the real world. Hand gestures not only play a complementary role in speech-based interaction but also express brain control behavior. Hand motion capture is significant in many HCI (human–computer interaction) applications, which can be used in virtual reality, intelligent driving, action recognition, and other industrial applications.

Hand pose estimation is essential for the above HCI applications by capturing gestures from videos or images. It has been studied in computer vision for decades [1,2] and the research focus has shifted from 2D hand pose estimation to 3D. Recent methods focus on inputting single RGB images or depth maps to estimate 3D hand poses [3,4,5,6,7,8,9]. Although these methods provide satisfactory results in some cases with challenging single-frame input, 3D hand pose estimation still suffers from the inherent depth ambiguity of the monocular setting, self-occlusions, background noise, and serious affect by light.

With the popularity of commodity RGB-D cameras, great performance in scene understanding, 3D reconstruction, and key-point detection applications have been achieved. Single depth-based methods suffer from overcoming inherent defects, such as depth information loss at the edge of the depth map, and thus single RGB-based methods can only provide rich semantic and texture information, rather than depth information. The fusion of RGB and depth information complements the two modalities, with the depth map providing the depth information that is missing from the RGB and RGB image, providing more features to offset the lack of edge information in the depth map. Despite the lack of studies in RGB-D-based hand pose estimation, the RGB-D fusion scheme has been explored for other tasks, among which semantic segmentation is the most extensively studied.

**Figure 1:**
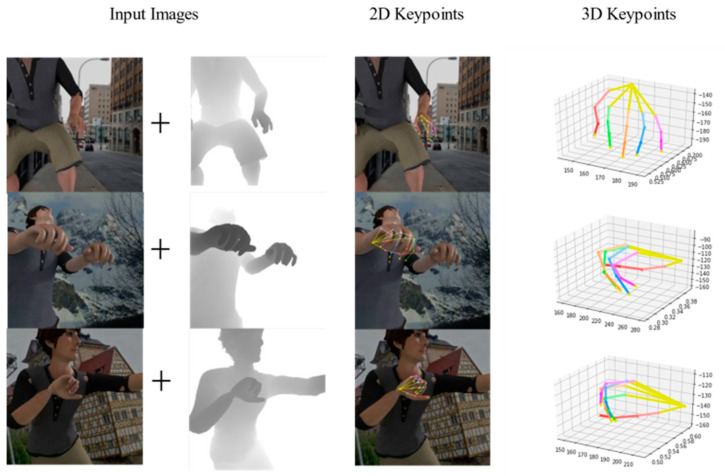
We propose a novel method (CrossFuNet) to fuse depth and RGB information from RGB images and paired depth maps. Our method is able to estimate 2D and 3D hand-joint locations. The first column shows the input frames, while the second column and third column show our 2D and 3D key-point results on the RHD dataset, respectively.

The benefits of the fusion of RGB and depth information, as well as the success of RGB-D in other similar fields, have prompted us to combine depth and texture information to obtain a more correct 3D hand pose. Therefore, we propose a novel end-to-end network (CrossFuNet) for the fusion of RGB and depth information, as shown in Figure 1. Our network consists of three parts: the initial feature extraction module, cross-fusion module, and 3D regression module. Our method firstly extracts RGB and depth feature maps using a two-stream initial feature extractor. For the RGB subnetwork, we chose ResNet50 [10] as the feature extractor because of its excellent ability to extract image features. As for the depth part, we chose ResNet18 [10] as the feature extractor. To better fuse RGB and depth feature information, as well as realize the complementary benefits of the two modalities, we designed a cross-fusion module in which the initial feature maps will be subjected to further feature extraction and information fusion. In the cross-fusion module, we obtained the final RGB-D feature map, which is used as input to the regression module to obtain the 3D joint coordinates. In the regression module, we can also derive 2D joint coordinates.

In summary, our main contributions include:

- Proposing an end-to-end RGB and depth input hand pose estimation network, which is called CrossFuNet, to achieve 2D and 3D hand pose estimation. We designed a two-stream initial feature extractor and cross-fusion module to obtain RGB and depth feature maps, and fuse them before the final regression module.
- Designing a novel RGB-D fusion method where the initial feature information is cross-fused with the refined features of the corresponding modalities. The RGB branch learns the texture, contour, and color information of the hand, while the depth branch learns the distance information, to ensure that the network can combine the two branches to better obtain 3D hand key-points.
- Our approach works well on challenging pubic datasets. Through extensive evaluations on two public datasets, we show that our proposed method can produce accurate and reasonable 3D hand poses.

The rest of the paper is organized as follows. Section 2 reviews the related works about the single input hand pose estimation. Section 3 describes our method in detail, while Section 4 shows our experimental results and some details. Finally, our paper concludes and future research direction in Section 5 and Section 6.

## 2. Related Work

We focus on RGB-D fusion methods, thus we will discuss methods according to the types of inputs. We will discuss methods based on depth, RGB, and RGB-D.

### 2.1. Depth-Based

Hand pose estimation employing depth maps as input is early because of the rise of Kinect [11]. However, due to the limitations of computing power and data capacity at that time, those previous works [4,5,6,12,13,14,15] are devoted to 2D pose estimation rather than 3D. Different from the above methods, Oberweger et al. [12] attempted to use shallow CNN to directly regress hand key-points. Now, the wide application of CNN has improved the accuracy of 3D hand pose estimation. Most of the works convert single-depth maps into 3D joint structures [16,17]. Chen et al. [18] introduced initial attitude estimation as guidance on the basis of the region ensemble (REN) network [19] proposed previously. The REN method extracted regions from feature maps and then concatenated before the FC (full connection) layer, which improved the accuracy compared with the regression key-points directly from the feature map. Yuan et al. [20] summarized and compared some state-of-the-art methods and put forward the challenges of depth maps. In this field, the lack of large datasets has always been a major problem for researchers. Individual researchers cannot afford the human, material, and financial resources required for large dataset shooting and calibration. There are some good solutions to this problem. Zhang et al. [21] proposed a two-stage network to augment training, which can improve the performance of small datasets. Baek et al. [22] used the generative adversarial network (GAN) to synthesize new data that have more angle characteristics by learning skeleton information, abandoning the previous method of using the depth map to generate confrontation data. Although these works achieve good results in some cases, depth-based methods still suffer from overcoming the inherent defects, such as depth information loss at the edge of the depth map. In addition, the depth cameras work really well in indoor scenes and have distance-limiting features, making depth-based approaches hard to scale in practice.

### 2.2. RGB-Based

Similar to the depth-based methods, the RGB methods are also studied from 2D to 3D perspectives. Toshev et al. [7] designed a CNN network to directly regress 2D joint positions. Tompson et al. [23,24] chose a discriminative method to regress score maps. As technology developed and research deepened, most studies tackled 3D hand poses using the deep-learning method with RGB inputs and achieved good results in some cases. The majority of 3D hand pose estimation methods [25,26,27,28] followed for a two-stage pipeline. First, they designed a feature detector to extract feature maps from an RGB input image and generated 2D heatmaps for each joint. Second, regression of 3D joints by a specific network that used 2D heatmaps as input was done. Zimmermann et al. [25] first solved the problem of 3D hand pose estimation with a learning-based formulation. Regressing 3D hand key-points from heatmap is challenging; these methods also suffer from depth ambiguity due to the absence of depth information. Cai et al. [26] proposed to leverage depth maps to get better hand pose estimation results during training, while testing only needs RGB images. Chen et al. [27] used a depth regularizer to regularize 3D hand poses during training. In contrast to the previous method, [27] selected a GAN network to generate depth maps to avoid entering pairs of RGB image and depth maps. Ge et al. [28] also leveraged the depth map as weak supervision in training but used a graph convolutional neural network (Graph CNN) as the feature extract network. All of the above approaches provide solutions to the challenges inherent in RGB-based approaches but weak monitoring is not as effective as the direct fusion of depth information. In other words, none of the above methods should be used in a dim setting.

### 2.3. RGB-D-Based

While there are few RGB-D-based hand pose estimation papers, we only found [29,30,31,32] which are most relevant to our work. Kazakos et al. [29] designed a two-stream architecture to extract color and depth information. RGB images and depth maps were fed into two separate nine-layer convolutional networks and then fused the feature maps before the FC layer. Different from [29,30], fused RGB images and depth maps to form a four-channel RGB-D image in the input layer. Furthermore, they used the REN network [19] to train the results. Mueller et al. [32] chose the same method to make a four-channel RGB-D input, which can be find in [30]. They designed a two-stage network to localize the hand and regress 3D joint locations. Specifically, the first stage of the architecture used a CNN to estimate the 2D position of the hand center in order to achieve hand localizing. Together with the cropped RGB-D image, the localized hand position was fed into a second CNN to regress relative 3D hand-joint locations in real-time. Zimmermann et al. [31] proposed an approach to estimate 3D human poses by designing a network to combine color images and depth maps. They predicted a 2D score map by color image detector and combined depth maps to regress 3D human poses.

Our method is different from the above methods. First, considering the difference between color and depth information, we designed two different subnetworks to extract their features instead of using the same network. Second, the fusion of color information and depth information occurs at the feature level rather than fusing into RGB-D at the input level. Third, we chose heatmaps to obtain 3D coordinates instead of directly regressing 3D hand poses.

## 3. Method

**Figure 2:**
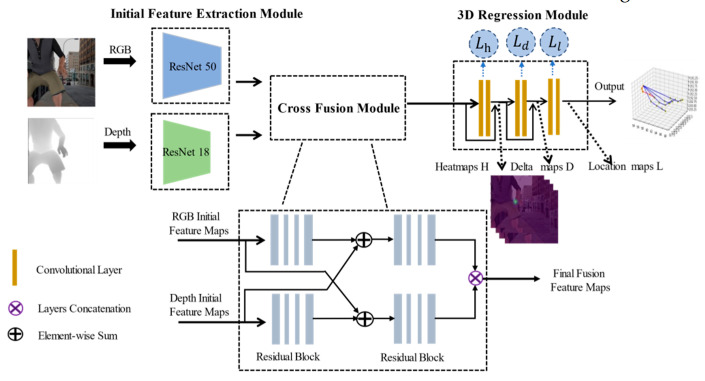
Overview of our architecture. It contains three modules: the *initial feature extraction module, cross-fusion module, and 3D regression module*. First, our initial feature extractor obtains initial RGB and depth feature maps from a pair of RGB images and depth maps. Second, the feature maps are fused in the cross-fusion module. Third, the regression module obtains both 2D and 3D hand-joints.

In order to compare with the previous works, we chose the popular 21 joint key-points. We designed a two-stream architecture to fuse RGB and depth feature maps, which is called CrossFuNet. An overview of the network is shown in Figure 2.

The goal of our network is to obtain joint positions both in 2D image space and 3D. We chose the common 3D hand-joint estimation architecture as our framework. Our network can be divided into three modules: *the initial feature extraction module, cross-fusion module, and 3D regression module*. For the RGB and depth initial feature extraction network, we designed two different architectures to extract feature information, respectively. Different branch networks are conducive to learning their own characteristics. The cross-fusion module consists of four residual blocks that we designed to fuse RGB and depth features better, and to output the final RGB-D feature maps. Finally, we obtained both 2D and 3D hand key-points in the 3D regression module using the common heatmap (location map) regression method. In the following sections, we will discuss our work in detail.

### 3.1. Initial Feature Extraction

We use RGB image $I_{c}$ and depth map $I_{d}$ to estimate 3D-space hand-joint positions $J={\left\{j_{i}\right\}}_{i=1}^{J}$, where $j_{i}=\left(x_{i},y_{i},z_{i}\right)$ and J is the number of key-points, i.e., i∈[1,J] with *J* = 21 in our case. The RGB image and depth map provide different feature information and should not be generalized with the same network. Therefore, unlike previous work [29], we designed a two-steam initial feature extraction network. The RGB image and its paired depth map are no longer input into the same network but rather into different architectures. In our network, the RGB branch provides more details such as regarding textures and colors. The depth branch provides high-level information such as location and depth. Although the RGB image can also provide hand position and high-level location information, the RGB image is susceptible to the background and does not work well in less-than-ideal lighting conditions. Thus, we gave the depth branch an expectation and chose a shallow network that is better for learning high-level features. The shallow network can effectively reduce the number of network parameters.

There have been many excellent works based on a single-RGB hand pose estimation sets, all of which have achieved good results and also contributed to their feature extraction network. Considering the stability of the initial feature extraction network and the convenience of comparing with our baseline [3], we chose to use the backbone of the ResNet50 as our RGB image initial feature detector. Since the convolution operation of the network will waste some information, the residual block in ResNet is equivalent to directly taking the previously processed information back for processing together, which plays a role in reducing information loss. For the depth feature extractor, we chose ResNet18 to extract more high-rise features such as location and depth. Considering that the output of the depth branch needs to be consistent with the RGB branch, we discarded part of the convolution layer.

Each branch takes images at a resolution of 128 × 128 and output feature maps at 32 × 32. Each branch receives its own primary feature maps F with a size of 32 × 32 × 256.

### 3.2. Multi-Cross-Fusion

There is a lack of extensive research on hand pose estimation based on deep learning RGB-D input and predecessors provided so few fusion methods of RGB images and depth maps. Previous works either directly concatenate the depth map, as the fourth channel of the RGB image, as the RGB-D input or designed two parallel feature extraction networks to fuse depth and RGB feature maps after the extraction module.

Our purpose for CrossFuNet is to better integrate the information of RGB images and depth maps. The simple fusion method cannot make the network learn RGB-D features better, thus we designed a cross-fusion network. Our fusion network aims to refine features and the cross-fusion between RGB and depth feature maps. Although the RGB image and depth map represent the same scene, they each have their own characteristics. RGB image feature information is rich, while depth maps can indicate distance. The RGB branch is used to learn the texture, contour, and color information of the hand, while the depth branch learns the distance information. Thus, the network can combine the two branches to better obtain 3D hand key-points. The features learned by the RGB branch can provide more information regarding the depth branch to help it learn the depth features better. The depth branch can also learn the contour and relative position of the hand, which can provide information supplements for the RGB branch. Cross-fusion of the two modalities can enhance the same characteristics and complement the information. If the fusion network is parallel, some features that complement and enhance each other will be lost. The cross-fusion network can provide more information for each sub-branch, which is more conducive to integrate RGB and depth features.

**Figure 3:**
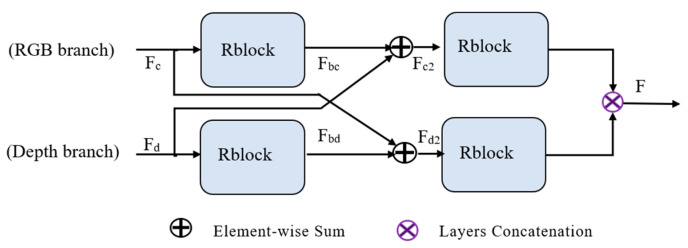
Overview of the cross-fusion module. Rblock denotes the residual block. F_c_ denotes the output of the RGB feature extraction branch and F_d_ is the output of the depth branch. F_c2_ and F_d2_ are the middle output of this module and F is the final output.

Therefore, we designed a novel fusion network, as depicted in Figure 3. We let the RGB branch and depth branch fuse with the corresponding branch in this module. The initial feature maps F_c_ and F_d_ are inputs to the residual block to extract more information into each branch. The output residual feature maps F_bc_ and F_bd_ will be added to the corresponding initial feature maps at the pixel level. In particular, F_c_ will add to F_bd_ and F_d_ will add to F_bc_. Thus, here we have the first combination of RGB and depth features: F_c2_ and F_d2_. In order to enable the network to better learn the fused features, we inputted the two fused features into the residual block and concatenated their feature maps as the final output F.

### 3.3. Regression Module

Our regression network, following the structure in [3], is mainly divided into two parts: the 2D detector and 3D detector. The 2D detector is a compact CNN architecture to generate heatmaps and 2D key-points coordinates can be obtained from the heatmaps. The 3D part generates 3D heatmaps called location maps from which we can derive the coordinates of the 3D key-points.

#### 3.3.1. 2D Detector

The 2D detector composed of a two-layer CNN takes fused RGB and depth feature maps F and generates the heatmaps H, containing 21 heatmaps with a size of 32 × 32. As in [24], the heatmaps encode the probability distribution of the possible position of each hand key-point. Heatmaps are crucial in our hand pose estimation because they are an essential part of the 3D detector and can regress 2D hand-joint locations.

#### 3.3.2. 3D Detector

The 3D detector inputs the feature maps F and heatmaps H to generate the final 3D hand point positions J=(X,Y,Z). Although the principle of the 3D detector is similar to that of the 2D detector, there are also differences in the details. In this part, we also introduce an additional D, namely the delta map, similar to [33]. The bone direction of each corresponding joint is encoded in D, which can provide extra kinematic information to make the 3D estimation more reasonable.

In the 3D detector, we first used a two-layer CNN to estimate the delta maps D from the heatmaps H and feature maps F. Then, the output delta maps D, together with feature maps F and heatmaps H, will be input into another two-layer CNN to obtain the final location maps L. The generation principle of L is similar to heatmaps H and each pixel encodes the 3D coordinates of joint J. Similar to [34], we took the maximum value for each joint J from the corresponding location map L as the 3D hand-joint positions $j_{i}=\left(x_{i},y_{i},z_{i}\right)$. The location maps L and delta maps D are supervised by 3D annotations.

### 3.4. Loss Function

During training, we minimize the following loss function ℒ:

(1) $$ℒ=\alpha {ℒ}_{h}+\beta {ℒ}_{l}+\gamma {ℒ}_{d},$$

where ℒ denotes the mean squared error (MSE) function, while α, β, γ are the trade-off coefficients used to balance each loss. In our experiments, α, β, and γ are set to be 100, 10, and 1, respectively. Our loss function combines multiple loss conditions: 2D heatmap loss ${ℒ}_{h}$, 3D location map loss ${ℒ}_{l}$, and delta map loss ${ℒ}_{d}$.

#### 3.4.1. 2D Heatmap Loss

The loss function calculates the difference between the ground-truth heatmaps and the predicted heatmaps. We add a ground-truth heatmap as a constraint to guide the network towards better feature extraction. Minimizing the loss can ensure that the predicted heat maps are closer to the ground-truth heat maps.

(2) $${ℒ}_{h}={∥H-H^{GT}∥}_{2},$$

where ${∥\cdot ∥}_{2}$ denotes the MSE loss function, H denotes the estimated heatmaps, and $H^{GT}$ represents ground-truth heatmaps. The public datasets we chose only provide the coordinates of joint points without heatmaps. We followed the method in [3] to generate heatmap $H_{j}^{GT}$ for joint j. We will initialized an initial heatmap with the same size as the estimated heatmap and conducted Gaussian filtering with sigma = 0.1 on the corresponding initial heat map based on the 2D joint coordinates provided by the ground-truth.

#### 3.4.2. 3D Location Map Loss

As for 3D location map loss, we used the same function as previous works [3,34]. The loss function calculates the difference between the ground-truth location maps and the predicted location maps, weighted with $H^{GT}$. As mentioned in [3], we were only interested in $x_{j}$, $y_{j}$ and $z_{j}$ from their respective maps; thus, the loss is weighted with $H^{GT}$.

(3) $${ℒ}_{l}=H^{GT}\;⊙\;{∥L-L∥}_{2},$$

where ⊙ is Hadamard product.

#### 3.4.3. Delta Map Loss


(4) $${ℒ}_{d}=H^{GT}⊙{∥D-D^{GT}∥}_{2},$$

The delta map loss function is similar to above, calculating the difference between the ground-truth delta maps and the predicted delta maps, weighted with $H^{GT}$.

## 4. Experiment

### 4.1. Implementation Details

We evaluated our approach on two public datasets: the Rendered Hand Dataset (RHD) [25] and the Stereo Hand Pose Tracking Benchmark (STB) [35].

The RHD dataset provides 41,258 training and 2728 testing samples. Each sample contains an RGB image, the corresponding depth map, mask image, 2D and 3D annotations, and camera parameters. In our case, we did not use mask images. As a synthetic dataset, RHD has many different hand shapes and various scenes, which make it a challenging dataset.

The STB dataset contains 12 sequences of a unique subject with 1800 frames in total. It contains two different subsets called SK and BB, and only the BB subset provides a corresponding depth map. We chose to use the SK subset and added the additional depth map SK_depth_seg to the dataset. We split the first scenario as our test dataset, namely B1Counting and B1Random, and the rest as our training dataset. The segmentation method of our training set and test set is consistent with the related papers [3,25,36]. Note that the STB dataset is a real scene dataset and the RHD is a synthetic dataset, thus they have different order of joint annotations. To compare the estimate results in an easier way, we aligned the joints in the STB and RHD to a new definition.

Earlier RGB-D-fusion papers have used the NYU dataset [23] but we did not use this dataset in our paper. The NYU dataset provides high-quality depth maps but their corresponding RGB images lack some information because it only provides color information for pixels with valid depth data.

We used the Adam optimizer [37] with the initial learning rate set at 1 × 10^−3^ and decayed at epoch 200 with decay rate of 0.1. We implemented it with PyTorch on NVIDIA TITAN Xp GPU. For both datasets, we cropped the input image to be centered at hand and resized it to the input resolution 128 × 128.

### 4.2. Evaluation Metric

There are two common evaluation methods in hand pose estimation: MPJPE and AUC. MPJPE measures the mean per joint position error in terms of the Euclidean distance (mm) between the prediction and ground-truth coordinates, as proposed in [18,38]. The error for the $j^{th}$ joint is calculated by:

(5) $$mpjpe_{j}=\frac{\sum i\left(∥k_{ij}-k_{ij}^{gt}∥\right)}{N},$$

where $k_{ij}$ denotes the network’s predicted hand key-point coordinates of the test frames; $k_{ij}^{gt}$ denotes the ground-truth hand key-point coordinates; N is the number of test frames; and J is the number of joints in a frame. The average joint error mpjpe is calculated by:

(6) $$MPJPE=\frac{\sum_{i}mpjpe_{i}}{J},$$

The AUC that we used reflects the success rate of the prediction, which is the area under the percentage of the correct key-point (PCK) curve (AUC), with thresholds ranging from 20 mm to 50 mm, as proposed in [3,26,39,40,41].

### 4.3. Comparison to Related Work

We compared our approach with other methods including weakly-supervised methods (Cai et al. [26] and Ge et al. [28]), depth-guided methods (Chen et al. [27]), and directly fused methods (Kazakos et al. [29]) on the RHD and STB datasets to illustrate the effectiveness of our proposed methods, as shown in Table 1 The results of the proposed methods (Cai et al. [26], Chen et al. [27], and Ge et al. [28]) on each datasets are obtained from their original papers. The results of Kazakos et al. [29] are re-implemented on the RHD and STB datasets. As shown in Table 1, our method (CrossFuNet) outperforms the other methods (Cai et al. [26], Chen et al. [27], Ge et al. [28], and Kazakos et al. [29]) on the RHD dataset, which denotes the promising performance of our proposed method.

For RHD, our method performs best. References [26,27,28] used depth information as a regularizer to obtain 3D hand key-points but our method used depth maps as input directly. The depth regularizer, as one of the weak supervision methods, is not as effective as the direct fusion of depth information. Reference [29] is the most similar method to our method but this method, which used nine-layer CNN, was too early to design complex architectures. On the RHD and STB datasets, the method in [29] used the nine-layer CNN architecture but could not obtain a good performance. Our method is not much better on STB than the other methods because the STB dataset has a smaller data volume and more uncomplicated actions. Although our method is not optimal on the STB dataset, it is more accurate than several similar methods. For the RHD, it is a very challenging dataset. Our method shows a better performance than the previous related methods, indicating that depth information added to challenging postures helps improve the accuracy of the results. The comparison with [29] proves that the fusion of different branch networks is beneficial to improve accuracy.

We compared our study to that of Kazakos et al. [29] because their approach is most similar to ours in that it used RGB-D as input for fusion at the feature level. Note that the data shown in Table 1 are all derived from the training results of our re-implemented network.

Note that the methods in [26,27,28] are originally trained and tested on the same datasets as ours. The method in [29] most relevant to our ideal method was originally trained on the NYU dataset. In order to make an intuitive comparison, we re-implemented their network using PyTorch and both trained and evaluated on the same dataset as ours. The experimental results of using the evaluation metrics are described in Section 4.2.

In practice, the depth camera is greatly affected by the environment and distance. We want our network to work well with only RGB input when we cannot obtain a depth map or when depth is not available. Therefore, we compared our network (w/o depth) with single-RGB input methods, as shown in Table 2 As our baseline, Zhou et al. [3] presented a clear 3D hand pose estimation architecture. Their method consisted of two parts, in which the first part was the 3D hand pose estimation and the second part was the output the hand mesh. Zimmermann et al. [25] followed a two-stage pipeline to obtain 3D key-points.

The data in Table 2 show that our method achieves good results even with only RGB input. This shows that our method can obtain better results by relying on RGB images when the depth sensor distance is far, which is important in practice because the quality of the input image is not always ideal. The distance of the structured light depth sensor is usually within 2 m. If our model wants to be used in long-distance situations, we need to rely on the RGB sensor to obtain images, such as long-distance human–computer interactions. To demonstrate that the fusion of RGB and depth helps improve network accuracy, we trained only the RGB branch on the same dataset.

In the methods listed in Table 1 and Table 2, “Ours” represents the network structure CrossFuNet proposed in our paper and “Ours (w/o depth)” represents the removing of the depth branch and only using the input the RGB network. We used the Adam optimizer [37] with the initial learning rate set at 1 × 10^−3^ and decayed at epoch 200 with the decay rate 0.1 to obtain our experimental results in Table 1 and Table 2. The networks were trained for a total of 500 epochs and the batch size was 128. Finally, the network’s implementation was based on Pytorch.

### 4.4. Ablation Study

**Figure 4:**
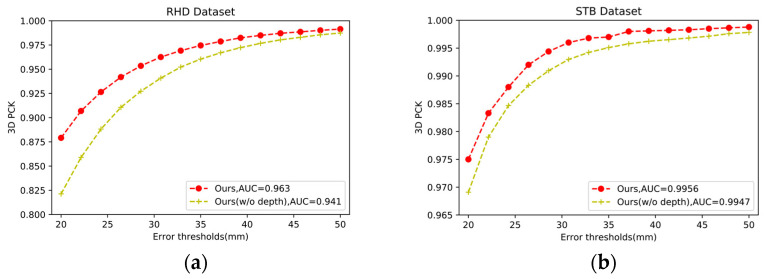
Ablation study for the training data on the RHD (**left**) and STB (**right**). (**a**) Describes the effectiveness of RGB image and depth map input in the RHD. (**b**) Describes the effectiveness of RGB image and depth map input in the STB.

**Figure 5:**
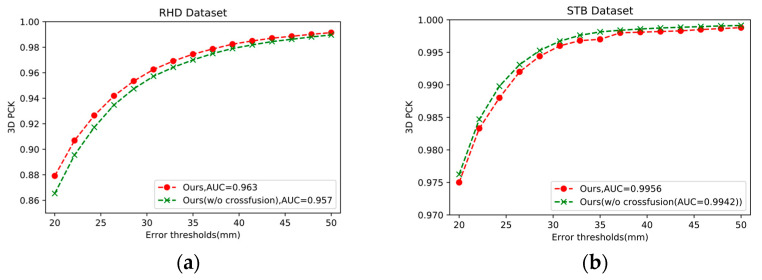
Comparative experiment of the effectiveness of the cross-fusion module on the RHD (**left**) and STB (**right**). (**a**) Describes the effectiveness of RGB image and depth map input in the RHD. (**b**) Describes the effectiveness of RGB image and depth map input in the STB.

We conducted an ablation study to better understand the impact of different components and input types in our network. We wanted to verify that combining RGB and depth information helps to improve the accuracy of the model; thus, we compared the whole RGB-D model (ours) without depth input (w/o depth) on the RHD and STB datasets. We compared the whole RGB-D module (ours) with the network (w/o cross-fusion) to verify the effect of the cross-fusion module on the RHD and STB datasets. Detailed experimental results are shown in Figure 4 and Figure 5.

#### 4.4.1. Effectiveness of RGB-D Input

To verify the superiority of RGB image and depth map input in the experiment, we compared the results of different inputs in the same network structure. Specifically, in order to ensure the rationality of the experiment, we reserved the residual block of the fusion module for the single-RGB input network.

As illustrated in Figure 4, *Ours* achieves an AUC of 0.963 within the error threshold 25–50 mm and ours (w/o depth) is 0.941 on the RHD dataset, which improves by 2.33%. On the STB dataset, although *Ours* is better than the ours (w/o depth) network, the improvement is not obvious. This is because the STB dataset has a small data capacity and is easily saturated. Depth information has obvious effects on complex gestures with occlusion but has little effect on simple gestures. From Table 3, we can observe that the error will decrease when we add the depth information. From another evaluation matric, MPJPE, we prove that the fusion ratio of RGB and depth improved only the input RGB effect. The bold numbers indicate optimal results.

#### 4.4.2. Effectiveness of Cross-Fusion Module

In this section, we verify the effectiveness of the cross-fusion module. We compared our whole architecture with the no-fusion network (w/o cross-fusion), which cut off the cross-fusion module and concatenated feature maps after the initial feature extraction module. To evaluate our cross-fusion module, we removed and trained it on the same dataset as the full network. The experiment results are shown in Figure 5 and Table 4.

As shown in Figure 5, *Ours* achieves an AUC of 0.963 within the error threshold 25–50 mm and the ours (w/o cross-fusion) is 0.957 on the RHD dataset, which improves by 0.63%. On the STB dataset, the cross-fusion module also does its job. From Table 4, the bold numbers indicate optimal results in MPJPE evaluation matric and the cross-fusion module effectively reduces the error. Experimental results show that the cross-fusion module helps to enhance the results of hand pose estimation. The features taught by the RGB branch can provide more information to the depth branch to help it better learn the depth features. The depth branch can also learn the contour and relative position of the hand, which can provide information supplements for the RGB branch.

In our results, one phenomenon is noteworthy: the results of the two methods in Table 3 (Ours (w/o depth)) and Table 4 (Ours (w/o cross-fusion)) are inconsistent with the performance of the two methods on the STB dataset and RHD dataset. The results without depth (0.9947) are better than the results with depth (0.9942) possibly for the following reasons: First, the STB dataset itself has a relatively small amount of data, with simple actions and which are easy to learn. In addition, the quality of the depth maps in the STB dataset is not as high as that of RHD, with some missing parts. Second, the function of the cross-fusion module is to better combine the depth information and RGB information, and realize the fusion of different levels of features, which is beneficial for network learning. In the absence of a cross-fusion module, the effect of fusing depth information on the easy-to-learn STB dataset is as good as the effect without depth. Third, the training data of our network fluctuates greatly on the STB dataset. We selected the intermediate value among the three ideal results and saved it. It is possible that in the process of our experiment, the value with relatively large fluctuations resulted in the present result. Due to the uncertainty of time and the initialization of the deep habit itself, we cannot guarantee consistent data from each training session. In our experiments, the data on the RHD dataset were more representative.

#### 4.4.3. Effectiveness of the Depth Network Layers

In our opinion, the depth subnetwork should be designed as a shallow CNN architecture because the depth map contains less information and provides higher level information in the RGB-D fusion. In our network, we expected that the information learned by the depth branch can enhance the learning effect in the ideal case of RGB images and depth can play an auxiliary role in the case of poor lighting. We needed to choose a suitable shallow network to meet the above requirements. In order to verify our idea and find the most appropriate depth subnetwork structure for our network, we designed an ablation study on the depth subnetwork layer number. On the premise that RGB remains unchanged, depth branches are ResNet50, 3-layer CNN, 6-layer CNN, and ResNet18. We changed the display order of the experiment to make the table cleaner.

As shown in Table 5, when we chose ResNet18 as the depth initial feature extractor, our architecture received the best result. According to our thinking, the shallow network will show better performance, while the performance of deep networks will not be significantly improved and may have overfitting. We first chose the three-layer CNN architecture, which was the shallowest network, to output the same size feature maps as the RGB branch and the network performed greatly. Then, we increased the depth branch to six layers but the experimental results were not satisfactory. The accuracy of the RHD dataset was improved by a small amount (only 0.1%) but the accuracy of the STB dataset was decreased. Simple stacking was not good for network learning but improved accuracy on the RHD dataset, encouraging us to explore the deeper network. Thus, in the next experiment, we used the ResNet18 network. We chose RseNet50 because there were too many network parameters, leading to learning over-fitting. According to the experimental results, ResNet18 showed the optimal effect on both RHD and STB datasets. The above experimental results confirmed our original idea.

### 4.5. Qualitative Result

**Figure 6:**
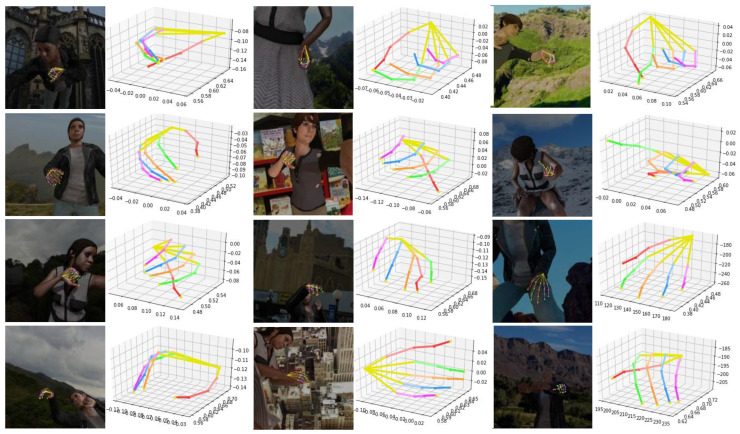
Some examples of our 2D and 3D results. The first, third, and fifth columns show the results of the 2D hand pose estimation. The second, fourth, and sixth columns show the results of the 3D hand pose estimation.

**Figure 7:**
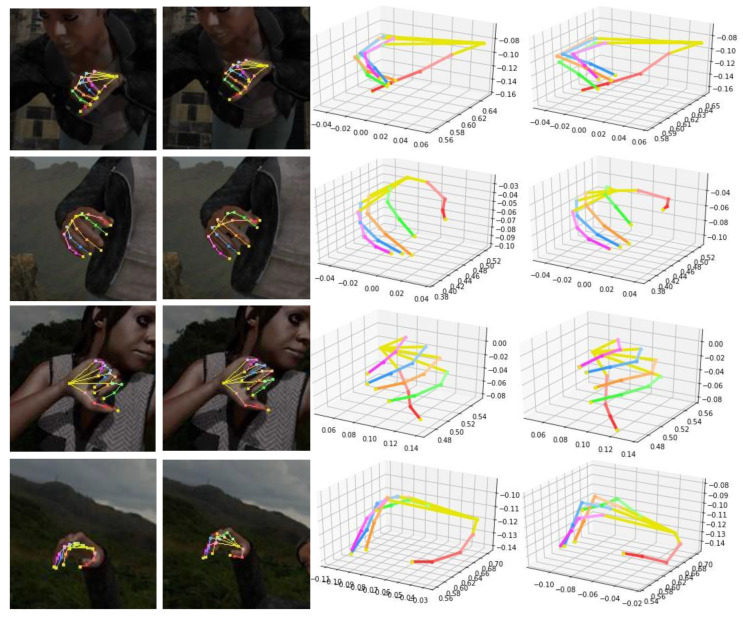

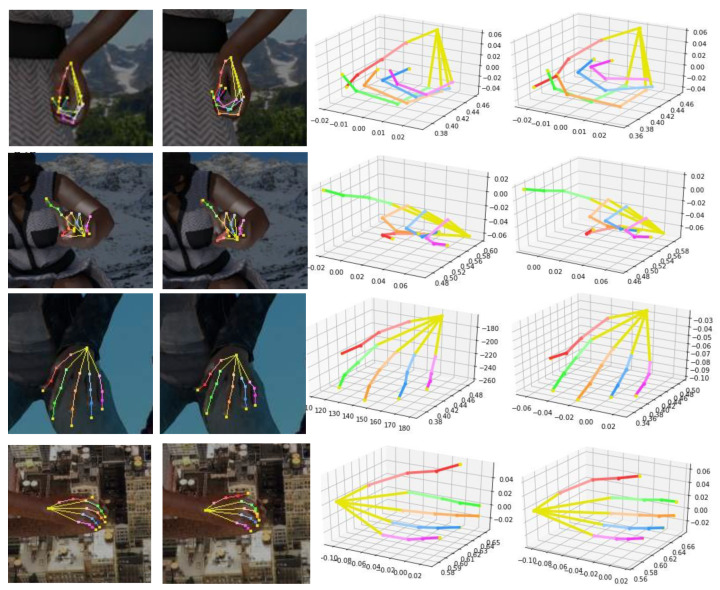
Comparison between the estimated 2D/3D key-points and ground-truth 2D/3D key-points on the RHD dataset. The first column shows our 2D results. The second shows the ground-truth 2D key-points. The third column is our 3D results and the fourth is the ground-truth 3D key-points.

Some results and comparation on the RHD dataset are shown in Figure 6 and Figure 7 We show the visualization 2D and 3D results of our proposed network in Figure 6. The first, third, and fifth columns show the results of 2D key-points, while the second, fourth, and sixth columns show the results of 3D key-points. Our network can accurately detect the key-points of gestures. The high accuracy of hand landmark localization is due to the addition of depth information, which helps to solve the challenge of occlusion. Considering the STB dataset is small and the action is simple, we only show the qualitative results of the RHD dataset, as shown in Figure 7. The first and fifth columns are our network’s 2D hand pose estimation results. The first column shows our 2D results. The second shows the ground-truth 2D key-points. The third column is our 3D results and the fourth is ground-truth 3D key-points. It is observed that the estimation of 3D hand poses is visually very similar to the ground-truth ones and for some self-occluded hand poses, as shown in Figure 6, our CrossFuNet also has a comparable estimated performance. Due to the complexity and challenges of the synthetic dataset RHD, as shown in Figure 7, while CrossFuNet’s predicted 3D hand posture is similar to the ground reality, there are still some gaps.

### 4.6. Discussion

Our method (CrossFuNet) proves that adding depth images to the input significantly improves the accuracy of hand pose estimation. Previous works [3,25] input only single RGB images, which cannot effectively solve the occlusion problem of 3D hand pose estimation. In papers [26,27,28], depth maps were used as additional information but the actual inputs were still RGB images, which is not ideal in the display scene. Our method directly input RGB images and depth maps to overcome the disadvantages of the above method. For the method in [29], although RGB images and depth maps were input as we did, they used the same subnetwork and the feature fusion method was simple. Our method uses different subnetworks in the RGB branch and depth branch, and presents an enlightening cross-fusion module. With the popularity of depth camera sensors, the acquisition of high-quality RGB-D images is becoming easier and easier. The RGB sensor captures high-definition color images and the depth sensor provides distance information. While previous methods need to work well under ideal conditions, our model can work well under poor light, under too much light, and beyond the limits of distance. Our method provides feasible ideas and solutions for intelligent home, intelligent monitoring, and in-car gesture recognition.

## 5. Future Work

Our network achieves ideal accuracy on the STB and RHD datasets but we do observe that the performance of the cross-fusion module is not significantly improved and the generalization capability of real-world data is poor. Considering the cross-fusion module we proposed previously is heuristic, improving our RGB and depth-fusion method is expected to directly improve our overall accuracy. The generalization capability of our network suffers from lacking real-word datasets. In the future, we plan to expand our cross-fusion module to improve the fusion effect of RGB and depth. We also plan to use the images captured by the depth camera to improve the generalization capability further. In addition, we will focus on the research of hand-motion recognition using the skeleton point outputs from our network.

## 6. Conclusions

The widespread popularity of consumer RGB-D cameras makes it easier to obtain RGB images and the corresponding depth maps, which has promoted the research progress of the RGB-D method in computer vision. However, there is a lack of corresponding fusion methods in hand pose estimation. Although 3D hand pose regression based on a single-RGB image or depth map is more challenging, it loses some information. In this paper, we proposed a novel RGB and depth feature-fusion method (CrossFuNet) for 3D hand pose estimation, which achieves better estimation results. By cross-fusion of the RGB and depth information, our method improves the feature-fusion effect. Our experiments prove that the fusion of RGB and depth information at the feature level can improve 3D hand pose estimation accuracy. Our method achieves ideal results on public RGB-D datasets.

## Figures and Tables

**Table 1:** Comparison with related works on the RHD and STB datasets. ‘-’ denotes that the original paper did not report this data. The bold numbers indicate optimal results.

Methods	AUC of PCK		MPJPE (mm)	
	STB	RHD	STB	RHD
Cai et al. [26]	0.9940	0.887	-	-
Chen et al. [27]	0.9900	0.859	9.11	19.0
Ge et al. [28]	**0.9980**	0.920	-	-
Kazakos et al. [29]	0.8730	0.820	-	-
Ours	0.9956	**0.963**	**7.03**	**10.09**

**Table 2:** Comparison with state-of-the-art single-RGB methods.

Methods	AUC of PCK	
	STB	RHD
Zhou et al. [3]	0.8980	0.856
Zimmermann et al. [25]	**0.9480**	^1^
Ours (w/o depth)	**0.9947**	**0.941**

^1^ We do not find this data in the original paper.

**Table 3:** Error analysis of input type effectiveness.

Network	AUC of PCK		MPJPE (mm)	
	STB	RHD	STB	RHD
Ours (w/o depth)	0.9947	0.941	7.24	11.50
Ours	**0.9956**	**0.963**	**7.03**	**10.09**

**Table 4:** Error analysis of the cross-fusion module effectiveness.

Network	AUC of PCK		MPJPE (mm)	
	STB	RHD	STB	RHD
Ours (w/o cross-fusion)	0.9942	0.957	7.27	10.95
Ours	**0.9956**	**0.963**	**7.03**	**10.09**

**Table 5:** Effectiveness of the depth network architecture.

Network	AUC of PCK		MPJPE (mm)	
	STB	RHD	STB	RHD
ResNet50	-	0.650 *	-	48.00 *
ResNet18	**0.9956**	**0.963**	**7.03**	**10.09**
3-layer CNN	0.9932	0.952	7.16	12.22
6-layer CNN	0.9927	0.953	7.22	12.36

* This data is overfitting.

## Data Availability

Data is contained within the article.
